# Effect of Manual Lymphatic Drainage on the Concentrations of Selected Adipokines, Cytokines, C-Reactive Protein and Parameters of Carbohydrate and Lipid Metabolism in Patients with Abnormal Body Mass Index: Focus on Markers of Obesity and Insulin Resistance

**DOI:** 10.3390/ijms241210338

**Published:** 2023-06-19

**Authors:** Klaudia Antoniak-Pietrynczak, Katarzyna Zorena, Marta Jaskulak, Rita Hansdorfer-Korzon, Marek Koziński

**Affiliations:** 1 Department of Immunobiology and Environment Microbiology, Medical University of Gdansk, Dębinki 7, 80-211 Gdansk, Poland; marta.jaskulak@gumed.edu.pl; 2 Department of Physiotherapy, Medical University of Gdansk, Dębinki 7, 80-211 Gdansk, Poland; rita.hansdorfer-korzon@gumed.edu.pl; 3 Department of Cardiology and Internal Diseases, Institute of Maritime and Tropical Medicine, Faculty of Health Sciences, Medical University of Gdansk, Powstania Styczniowego 9b, 81-519 Gdynia, Poland; marek.kozinski@gumed.edu.pl

**Keywords:** overweight and obese patients, manual lymphatic drainage, biochemical parameters, leptin, adiponectin, IL-10, VEGF, cut-off value

## Abstract

The aim of the study was to assess the impact of manual lymphatic drainage (MLD) on the parameters of carbohydrate metabolism, lipid metabolism and the level of selected adipokines and cytokines in people with abnormal body mass index (BMI). In addition, an attempt was made to assess the optimal cut-off values of serum concentrations of the biochemical parameters studied in identifying the risk of obesity and insulin resistance (IR). The study included 60 subjects who underwent 10 and 30 min long MLD sessions three times a week. The study group included 15 patients with a normal body mass index (group I; *n* = 15), overweight patients (group II; *n* = 15) and obese patients (group III; *n* = 10). The control group was IV; *n* = 20 subjects not undergoing MLD. Biochemical tests were carried out on all subjects at stage 0′ (before MLD therapy) and at stage 1′ (one month after MLD therapy). In the control group, the time between the sample collection at stage 0′ and stage 1′ was the same as in the study group. Our results showed that 10 MLD sessions may have a positive effect on the selected biochemical parameters, including insulin, 2h-PG, leptin and HOMA-IR values in normal weight and overweight patients. In addition, in the study group, the highest AUC_ROC_ values in identifying the risk of obesity were found for leptin (AUC_ROC_ = 82.79%; cut-off = 17.7 ng/mL; *p* = 0.00004), insulin (AUC_ROC_ = 81.51%; cut-off = 9.5 µIU/mL; *p* = 0.00009) and C-peptide (AUC_ROC_ = 80.68%; cut-off = 2.3 ng/mL; *p* = 0.0001) concentrations as well as for HOMA-IR values (AUC_ROC_ = 79.97%; cut-off = 1.8; *p* = 0.0002). When considering the risk of IR, we observed the highest diagnostic value for insulin (AUC_ROC_ = 93.05%; cut-off = 1.8 ng/mL; *p* = 0.053), which was followed by C-peptide (AUC_ROC_ = 89.35%; cut-off = 17.7 ng/mL; *p* = 0.000001), leptin (AUC_ROC_ = 79.76%; cut-off = 17.6 ng/mL; *p* = 0.0002) and total cholesterol (AUC_ROC_ = 77.31%; cut-off = 198 mg/dL; *p* = 0.0008). Our results indicate that MLD may have a positive effect on selected biochemical parameters, including insulin, 2h-PG, leptin and HOMA-IR, in normal weight and overweight patients. In addition, we successfully established optimal cut-off values for leptin in the assessment of obesity and insulin in the assessment of insulin resistance in patients with abnormal body mass index. Based on our findings, we hypothesize that MLD, when combined with caloric restriction and physical activity, may serve as an effective preventive intervention against the development of obesity and insulin resistance.

## 1. Introduction

The World Health Organization [1] defines obesity as a complex chronic disease in which an excess or dysfunction of adipose tissue poses a health risk [2]. The global prevalence of obesity, which currently affects more than 650 million people, has tripled in the last 50 years. Overweight, which in turn is a precursor to obesity, now affects more than 25% of the world’s population, or 1.9 billion people [3]. According to the current state of knowledge, obesity is strongly associated with the development of insulin resistance (IR) [4]. Insulin resistance, in turn, plays a key role in the pathogenesis of cardiometabolic complications, including components of the metabolic syndrome, hypertension, atherosclerosis, and type 2 diabetes mellitus (T2DM) [4,5,6,7]. Insulin resistance is characterized by a reduced ability of insulin to stimulate glucose uptake by adipose and muscle tissue, as well as to inhibit glucose synthesis and production in the liver [8]. In addition, increased basal lipolysis in adipose tissue and elevated levels of circulating free fatty acids (FFA) are observed in obesity [9]. An excessive supply of FFA in the later period may, in turn, negatively affect glucose transport to skeletal muscles and inhibit insulin activity [10,11]. In all forms of obesity, there is also a decrease in the level of glucose transporter type 4 (GLUT4), which is the main factor affecting the impairment of insulin-stimulated glucose transport in adipocytes. This, then, leads to the reduced disposal of glucose in adipose tissue [12]. In the light of new research, it is known that in addition to fasting plasma glucose (FPG), glycated hemoglobin (HbA1c), insulin, C-peptide and homeostatic model assessment–insulin resistance (HOMA-IR), the 2h-post-load glucose (2h-PG) level test is recognized as the most sensitive tool for assessing glucose metabolism [13,14,15,16,17,18]. In addition to carbohydrate metabolism disturbances, obese patients are also diagnosed with lipid metabolism disorders [19,20,21,22]. Studies have shown that the higher the body mass index (BMI), the higher the risk of dyslipidemia. Approximately 60–70% of obese patients present with dyslipidemia, while dyslipidemia in overweight patients is approx. 50–60% [19,20]. In addition to carbohydrate-lipid metabolism disorders, a characteristic of obesity is low-grade inflammation expressed by an increased value of high-sensitivity C-reactive protein (hsCRP) in serum [23], as well as proinflammatory cytokines, adipokines and growth factors [7,24,25]. For example, the available studies have shown that the development of inflammation and reduced insulin sensitivity in the course of obesity are associated with reduced levels of adiponectin [24,26,27]. Leptin, on the other hand, produced by the adipose tissue, is currently considered a satiety hormone and regulates blood glucose levels and processes related to appetite [24,28]. Recent studies have shown that circulating leptin levels are elevated in obese patients, suggesting a link between obesity and postoperative lymphedema. It is believed that the compression of the lymphatic vessels by adipocytes or fibrosis of the smooth muscles in the lymphatic system causes obesity-related lymphedema [28]. Vascular endothelial growth factor (VEGF) in physiological conditions is a key factor in maintaining normal endothelial function. However, in pathological conditions, its high concentration may cause abnormal angiogenesis [29]. Elevated VEGF levels are a key indicator of cardiovascular disease and are considered a risk factor for cardiovascular disease [29,30]. Moreover, Elias et al. showed that the overexpression of VEGF can cause decreased insulin sensitivity [31].

Various therapeutic strategies are used to prevent insulin resistance and obesity [6,14,26,32]. Both we and a few other researchers have shown that MLD can be a beneficial therapy for preventing the development of obesity, improving selected clinical parameters, and thus improving the quality of life of patients with an abnormal body mass index [14,32,33]. The use of MLD can support the flow of nutrients transported by the blood to the tissues, as well as improving the metabolism of adipose tissue and the removal of waste products [32].

The aim of this study was to assess the impact of MLD on the parameters of carbohydrate metabolism, lipid metabolism and the level of selected adipokines and cytokines in people with abnormal body mass index. In addition, an attempt was made to assess the cut-off values of serum concentrations of the biochemical parameters studied in identifying the risk of obesity and IR development in all subjects.

## 2. Results

### 2.1. Clinical Characteristics of Patients with Normal Body Mass Index (Group I), Overweight Patients (Group II), Obese Patients (Group III) and Controls (Group IV)

Ultimately, 60 subjects were included in the study, and the age of the study participants was 39 ± 11 years. The study group included 40 patients: patients with normal body mass index as group I (*n* = 15), overweight patients as group II (*n* = 15) and obese patients as group III (*n* = 10). The control group (IV) included 20 subjects. The age of the study participants was 39 ± 13 years on average in group I, 39 ± 11 years in group II, 39 ± 11 years in group III, and 39 ± 12 years in group IV. Group I patients had a statistically significantly lower BMI compared to the BMI of group II patients (*p* = 0.018) and compared to the BMI values of group III patients (*p* = 0.015). No statistically significant differences in the BMI level were detected between group I and group IV. Also, a statistically significantly lower visceral adipose tissue (VAT) value was detected in group I patients compared to the VAT value of group II patients (*p* = 0.002) and compared to the VAT value of group III patients (*p* = 0.005). No statistically significant differences in VAT values were found between group I and group IV. Moreover, group I was characterized by a statistically significantly lower level of the WHR index compared to group II (*p* = 0.00001) and compared to group III (*p* = 0.0000003). There were no significant differences in the WHR level between group I and group IV. In addition, significantly lower systolic blood pressure was detected in group I compared to systolic blood pressure in group III (*p* = 0.000000007). No statistically significant differences in the level of systolic blood pressure were detected between group I and group II and between group I and group I and IV. Patients in group II had a statistically significantly lower level of BMI compared to the level of BMI in patients in group III (*p* = 0.0001), and a statistically significantly higher level compared to the level of BMI in patients in group IV (*p* = 0.002). Group II patients had a statistically significantly lower VAT value compared to group III patients (*p* = 0.008). No statistically significant differences in the value of VAT were found between patients in group II and patients in group IV. Group II was characterized by a statistically significantly lower WHR level compared to group III (*p* = 0.0000002) and a statistically significantly higher WHR level compared to group IV (*p* = 0.00009). A statistically significantly lower level of systolic blood pressure was also detected in group II compared to group III (*p* = 0.0006), with no differences in systolic blood pressure between group II and group IV. Group III patients showed a statistically significantly higher BMI level (*p* = 0.000000005), VAT value (*p* = 0.0003), WHR level (*p* = 0.00000009), and systolic blood pressure value (*p* = 0.00000008) compared to group IV patients. There were no statistically significant differences between the groups in terms of parameters such as age and diastolic blood pressure. The clinical characteristics of the subjects are presented in Table 1.

### 2.2. Analysis of the Relationship between Biochemical Parameters and Anthropometric Indicators (BMI, WHR, VAT) of Patients in the Study Group and Those in the Control Group

The aim of the next stage of our work was to study the relationship between bio-chemical parameters and anthropometric indices (BMI, WHR, VAT) in the patients of the study group and in the control group. The correlations were performed at stage 0′ (before MLD therapy). The relationship between biochemical parameters and anthropometric indicators identified was due to differences in BMI, WHR, VAT. The results are presented in Table 2.

#### 2.2.1. Correlations between Biochemical Parameters and the BMI in the Study and Control Groups

In patients of the study group and the control group, a statistically significantly positive relationship was found between the BMI value and the concentration of 2h-PG (*r* = 0.321, *p* = 0.043), insulin concentration (*r* = 0.465, *p* = 0.002), C-peptide concentration (*r* = 0.471, *p* = 0.002), HOMA-IR (*r* = 0.433, *p* = 0.005), total cholesterol (*r* = 0.999, *p* = 0.001), LDL-C (*r* = 0.990, *p* = 0.001), VEGF (*r* = 0.998, *p* = 0.0002) and leptin concentration (*r* = 0.718, *p* = 0.0000001), and a statistically significant negative relationship between BMI and HDL-C concentration (*r* = −0.325, *p* = 0.040) was found.

No statistically significant correlations between the BMI and the concentration of FPG, HbA1c, hsCRP, TG, IL-10 and adiponectin concentration were found in the patients of the study group and in the control group.

#### 2.2.2. Correlation between Biochemical Parameters and the WHR of Patients in the Study Group and the Control Group

In the patients of the study group and the control group, a statistically significantly positive relationship was found between WHR and insulin concentration (*r* = 0.433, *p* = 0.005), C-peptide concentration (*r* = 0.447, *p* = 0.003), HOMA-IR value (*r* = 0.462, *p* = 0.002), hsCRP level (*r* = 0.779, *p* = 0.004), total cholesterol level (*r* = 0.909, *p* = 0.018), LDL-C level (*r* = 0.709, *p* = 0.018), TG level (*r* = 0.316, *p* = 0.046) and VEGF concentration (*r* = 0.716, *p* = 0.059) and between WHR and leptin concentration (*r* = 0.733, *p* = 0.00000007).

No statistically significant correlations between WHR and the concentration of FPG, 2h-PG, HbA1c, HDL-C, IL-10 and adiponectin concentration were found in the patients of the study group and the control group.

#### 2.2.3. Correlations between Biochemical Parameters and the VAT Value in the Study and Control Groups

In patients of the study group and the control group, a statistically significantly positive relationship was found between VAT and FPG concentration (*r* = 0.493, *p* = 0.001), 2h-PG concentration (*r* = 0.434, *p* = 0.005), HbA1c concentration (*r* = 0.444, *p* = 0.004), insulin concentration (*r* = 0.396, *p* = 0.005), C-peptide concentration (*r* = 0.322, *p* = 0.042), HOMA-IR value (*r* = 0.410, *p* = 0.008), total cholesterol concentration (*r* = 0.410, *p* = 0.008), *r* = 0.757, *p* = 0.050), LDL-C concentration (*r* = 0.757, *p* = 0.017), TG concentration (*r* = 0.500, *p* = 0.001), VEGF concentration (*r* = 0.868, *p* = 0.027), leptin concentration (*r* = 0.581, *p* = 0.00005) and adiponectin concentration (*r* = 0.810, *p* = 0.039). We also detected a statistically significantly negative relationship between VAT and HDL-C concentration (r = −0.490, *p* = 0.001). No statistically significant relationships were detected in patients in the study group and the control group between the value of VAT and the concentration of hsCRP and the concentration of IL-10.

### 2.3. Cut-Off Values of the Analyzed Biochemical Parameters in Identifying the Risk of Obesity in Patients in the Study and Control Groups

To investigate which of the 15 tested biochemical parameters (FPG, 2h-PG, HbA1c, insulin, C-peptide, HOMA-IR, total cholesterol, HDL-C, LDL-C, TG, hsCRP, VEGF, IL-10, adiponectin and leptin) showed the highest discriminant value in identifying the risk of obesity, we used the ROC (Relative Operating Characteristic) curve analysis. The ROC curve analysis calculations were performed at 0′ (before MLD therapy). In the case of obesity, we divided all the subjects into two groups: those with a BMI above 30.0 kg/m^2^ and those with a BMI below 30 kg/m^2^ (assuming BMI 30.0 kg/m^2^ or higher falls within the obesity range).

The highest AUC_ROC_ values identifying obesity were found for insulin, C-peptide, leptin and HOMA-IR values. The area under the ROC (AUC_ROC_) for insulin was found to be 81.51%, and its population value was within the range of 69.23–93.79%. The sensitivity was determined as 62.13% and the specificity as 32.45%, and the cut-off value for insulin was 9.5 µIU/mL. In patients in the study group and in the control group, the cut-off value for C-peptide was 2.3 ng/mL, the area under the ROC curve was 80.68%, and its population value was within the range of 68.73–92.62%. The sensitivity for the C-peptide was found to be 64.53% and the specificity 42.39%. Also, in all patients (study and control group), the threshold value for leptin was 17.7 ng/mL, the area under the ROC curve for leptin was 82.79%, and its population value was within the range of 71.51–94.08%; the sensitivity was 27.22% and specificity 41.92%. For HOMA-IR, the cut-off value in identifying the risk of obesity was 1.8, the area under the ROC curve for HOMA-IR was 82.79%, and its population value was within the range of 67.16–92.78%; the sensitivity was 69.53% and specificity 33.16%. The strongest predictors among the biochemical parameters of obesity risk were insulin and leptin. It was shown that the ROC curve for insulin lies below the ROC curve for leptin, and thus leptin was the indicator with the highest discriminant value for obesity. The difference in areas under the curve for insulin and leptin was *p* = 0.01. The results are presented in Table 3.

### 2.4. Cut-Off Values for the Analyzed Biochemical Parameters in Identifying the Risk of Insulin Resistance in Patients from the Study and Control Groups

To investigate which of the 14 parameters (FPG, 2h-PG, HbA1c, insulin, C-peptide, total cholesterol, HDL-C, LDL-C, TG, hsCRP, VEGF, IL-10, adiponectin and leptin) showed the highest discriminant value in identifying IR risk in patients from the study and control groups, ROC curve analysis was used. The ROC curve analysis calculations were performed at 0′ (before MLD therapy). In the case of insulin resistance, we divided all the subjects into two groups: those with a HOMA-IR index above 2.0 and those with a HOMA-IR index below 2.0 (according to the following formula: HOMA-IR = (FPG × fasting serum insulin)/405, assuming <2 as normal in adults).

The highest AUC_ROC_ value identifying IR was found for the concentrations of insulin, C-peptide, total cholesterol and leptin. The area under the ROC (AUC_ROC_) for insulin was found to be 93.05%, and its population value was within the range of 85.15–100%. The sensitivity was 78% and the specificity 36.9%, and the cut-off value for insulin was 6.9 µIU/mL. In patients in the study group and in the control group, the cut-off value for C-peptide was 1.8 ng/mL, the area under the ROC curve was 89.35%, and its population value was within the range of 78.93–99.77%. The sensitivity for the C-peptide was found to be 77.56% and the specificity 40.79%. Also, in all patients (study and control group), the cut-off value for total cholesterol was 198 mg/dL, the area under the curve for total cholesterol was 77.31%, and its population value was within the range of 63.82–90.80%; the sensitivity was 42.48%, and the specificity 67.75%. The IR risk cut-off for leptin was 17.6 ng/mL, the ROC for leptin was 79.76%, and its population value was within the range of 66.30–93.21%; sensitivity was 69.44% and specificity 39.77%. The strongest predictors among the biochemical parameters of IR risk were insulin, C-peptide and leptin. It has been shown that the ROC curve for leptin lay under the ROC curve for insulin and the ROC curve for C-peptide. The difference in areas under the curve for insulin and leptin was *p* = 0.133, and for C-peptide and leptin *p* = 0.095, whereas the difference in areas under the curve for insulin and C-peptide was *p* = 0.03; thus, insulin was the indicator with the highest discriminant value for the development of IR. The results are presented in Table 4.

### 2.5. Evaluation of the Impact of 10 MLD Sessions on Biochemical Parameters at Stages 0′ and 1′ in the Group of Patients with a Normal Body Mass Index (Group I), Overweight Patients (Group II), Obese Patients (Group III) and Control IV (without MLD Therapy)

In the group of patients with a normal body mass index (group I) after 10 sessions of MLD therapy, a statistically significant decrease in insulin level was detected from 8 µIU/mL at stage 0′ to 5 µIU/mL at stage 1′, i.e., 30 days after MLD therapy (*p* = 0.039). In addition, there was a statistically significant decrease in HOMA-IR, from 2 at stage 0′ to 1.1 at stage 1′ (*p* = 0.003) and a statistically significant decrease in leptin from 9.6 ng/mL at stage 0′ to 6.8 ng/mL at stage 1′ (*p* = 0.041). In group I, no significant variable differences were found between stage 0′ and stage 1 in terms of the levels of FPG, 2h-PG, HbA1c, C-peptide, total cholesterol, HDL-C, LDL-C, TG, hsCRP, VEGF, IL-10 and adiponectin. In the group of overweight patients (group II), after 10 sessions of MLD therapy, a statistically significant decrease in the level of 2h-PG was observed from 119 mg/dL at stage 0′ to 100 mg/dL at stage 1′ (*p* = 0.049). In addition, we observed a statistically significant decrease in HOMA-IR from 2.6 at stage 0′ to 1.9 at stage 1 (*p* = 0.049), and a statistically significant decrease in leptin concentration from 18.1 ng/mL at stage 0′ to 13.0 ng/mL at stage 1′ (*p* = 0.033). In group II, no statistically significant differences were found between baseline and stage 1 in terms of the level of FPG, HbA1c, insulin, C-peptide, total cholesterol, HDL-C, LDL-C, TG, hsCRP, VEGF, IL-10 and adiponectin. In the group of obese patients (group III) after 10 sessions of MLD therapy, a decrease was detected in the HOMA-IR index from 2.8 at stage 0′ to 2.0 at stage 1 (*p* = 0.169) (although it was statistically insignificant), and a decrease in leptin from 28.0 ng/mL at stage 0′ to 21.7 ng/mL at stage 1′ (*p* = 0.206). In addition, in the group of obese patients (group III), after 10 sessions of MLD therapy, no statistically significant differences were found between stage 0′ and stage 1′ in terms of FPG, 2h-PG, HbA1c, insulin, C-peptide, cholesterol total, HDL-C, LDL-C, TG, hsCRP, VEGF, IL-10 and adiponectin. In the control group (group IV), in which MLD therapy was not used, no statistically significant differences were found between stage 0′ and stage 1′ in terms of the level of FPG, 2h-PG, HbA1c, insulin, C-peptide, HOMA-IR, total cholesterol, HDL-C, LDL-C, TG, hsCRP, VEGF, IL-10, leptin and adiponectin. Biochemical parameters in the groups of patients with a normal body mass index, overweight patients and obese patients and in the control group at stage 0 compared to stage 1 are presented in Table 5.

## 3. Discussion

A key result of our research was that we detected cut-off values for leptin and insulin, which may potentially be additional predictors of insulin resistance and obesity. One of the statistical methods used to establish the predictive value and cut-off values of the parameters under study was ROC curve analysis [34,35]. The analysis of ROC curves for obesity showed that in all our patients (study and control), out of 15 tested parameters (FPG, 2h-PG, HbA1c, insulin, C-peptide, HOMA-IR, total cholesterol, HDL-C, LDL-C, TG, hsCRP, VEGF, IL-10, adiponectin, leptin), the highest discriminating powers for obesity were found for insulin, C-peptide, HOMA-IR and leptin. In the presented study, using the AUCROC analysis for obesity, we showed an insulin cut-off of 9.5 µIU/mL, a C-peptide cut-off of 2.3 ng/mL and a HOMA-IR cut-off of 1.8. However, in our study, the highest area under the curve in identifying obesity risk was found for leptin concentration. In the studied groups, the cut-off value for leptin was 17.7 ng/mL, and the area under the ROC curve for leptin was found to be 82.79%. For IR, the highest discriminating power was found for insulin, C-peptide, total cholesterol and leptin. The highest value of the area under the curve (93.05%) was found for insulin, and its cut-off value was 6.9 µIU/mL. In addition, we found a C-peptide cut-off of 1.8 ng/mL, a total cholesterol cut-off of 198 mg/dL and a leptin cut-off of 17.6 ng/mL. To the best of our knowledge, we are the first researchers to present a cut-off value for leptin towards IR development. In relation to the results of other research, our cut-off values were at the upper cut-off of reference values [36,37]. In the case of leptin, several researchers have already suggested that its measurement in serum may be useful in identifying the risk of obesity and/or IR, but there are still no clear definitions of reference values for its level [38,39,40]. As pointed out by Takeda et al., plasma leptin levels in healthy individuals should be in the range of 2.5–21.8 ng/mL [41], while researchers from the team of Sato et al. indicate that in obese and/or IR subjects, elevated leptin levels range from 10 to 100 ng/mL [28]. Under normal conditions, leptin, through negative feedback, reduces insulin secretion and increases insulin sensitivity, leading to the uptake of glucose for energy use or storage [42]. However, in the course of obesity and/or IR in a state of hyperleptinemia, the dysregulation of the adipocyte–insulin axis in pancreatic beta cells promotes hyperinsulinemia [43,44]. In turn, the action of insulin stimulates adipogenesis, thus leading to a further increase in insulin secretion, and consequently causes IR with the development of T2DM [43,44]. Research suggests that insulin and C-peptide levels are interdependent, and elevated levels of these parameters may promote atherogenesis, potentially increasing the risk of cardiovascular disease in people with obesity [15]. At the same time, in our group of subjects, we observed a significant positive correlation between BMI, WHR and VAT and leptin, insulin, C-peptide and HOMA-IR levels. Our results are consistent with those from previous studies by other researchers [45,46,47]. Additionally, in our study, we demonstrated the predictive value of the anti-inflammatory cytokine IL-10 for obesity (0.35 pg/mL), but not IR. In the study by Subramanian et al. [48] on obese patients without severe metabolic complications, IL-10 levels have been shown to be positively correlated with the amount of white adipose tissue (WAT) [48]. Other authors have previously suggested that the measurement of serum FPG, HbA1c, HDL-C, LDL-C, VEGF and adiponectin levels may be useful in identifying the risk of obesity and/or IR [49,50,51]. Our results do not confirm previous reports by other authors. One reason may be that T2DM patients were excluded in our study. Thus, we suggest that abnormal levels of these parameters may be a consequence of obesity rather than its direct cause. In the patients of the study group and in the control group, the level of adiponectin concentration was within the reference range [52]. Although other researchers reported a negative relationship between the concentration of adiponectin and indicators of obesity [26,27], in our study we did not manage to show a relationship between the concentration of adiponectin and the BMI and WHR in patients in the study. Paradoxical hyperadiponectinemia in metabolically healthy obese individuals has been reported by Ahl et al. [53]. Ahl et al. showed that the reduced secretion of adiponectin is mainly associated with abdominal obesity, while it is observed to a lesser extent in patients with increased amounts of subcutaneous fat [53]. Similarly, in the case of VEGF, a growth factor that can induce angiogenesis and lymphangiogenesis in vascular endothelial cells under both physiological and pathological conditions [29,31], we did not detect a prognostic value for obesity and IR. The lack of a prognostic value of VEGF was previously explained by Sun et al., who suggested that an increase in concentration in the early stages of diabetes and with impaired glucose tolerance may have a beneficial compensatory effect [29]. We suggest that increased VEGF expression may be both a response and a cause of disease exacerbation. Finally, in the next step of our study, we attempted to assess the biochemical parameters at stage 0′ (before MLD therapy) compared to stage 1′ (one month after MLD therapy) in patients of the study group (groups I, II and III) and at stage 0′ compared to stage 1′ (without MLD therapy) in patients in the control group (group IV). In patients from the control group, we did not detect statistically significant differences in any of the biochemical parameters tested at stage 0′ compared to the same parameters tested at stage 1′. In the patients of the study group, we did not find statistically significant differences in the concentration of FPG, HbA1c, C-peptide, hsCRP, total cholesterol, HDL-C, LDL-C, TG, VEGF, IL-10 and adiponectin. However, we did find statistically significant differences in the concentration of 2h-PG, insulin, HOMA-IR and leptin at stage 0′ compared to the tested parameters at stage 1′. Therefore, in group II patients (study group, overweight patients), we detected a statistically significant decrease in 2h-PG concentration by 19 mg/dL, from 119 mg/dL at stage 0′ to 100 mg/dL at stage 1′.

These results are consistent with the results of our previous study [32], which showed that 10 × 30 min sessions of MLD significantly contributed to a decrease in 2h-PG levels (by 12 mg/dL) in overweight patients. To our knowledge, in the available literature no other studies have analyzed the effect of MLD on the level of 2h-PG in people with abnormal body weight [32]. Despite different therapeutic techniques being applied, a similar effect on the level of 2h-PG was demonstrated by Xie et al. [54]. The researchers assessed the effect of abdominal massage on the concentration of 2h-PG in people with T2DM [54]. Reducing the level of 2h-PG by reducing the level of oxidative stress and inflammatory markers may contribute to reducing the risk of obesity consequences [55,56]. As early as the beginning of the 21st century, Brownlee et al. suggested that postprandial hyperglycemia plays a role in excessive production of reactive oxygen species [57]. Förstermann et al. showed that the overproduction of reactive oxygen species may promote the development of IR by inhibiting the activation of protective adiponectin and endothelial nitric oxide synthase (eNOS) [58]. The second parameter analyzed by us, in which we observed a decrease after the use of MLD, was insulin. In all subjects, the insulin concentration was within the reference range at both stage 0′ and 1′. However, as we showed in the IR predictive assessment, the IR cut-off for insulin in our subjects was 6.9 µIU/mL. In our study, after MLD in group I patients (patients with normal body mass index), we detected a statistically significant decrease in insulin levels below the cut-off value, from 8 µIU/mL at stage 0′ to 5 µIU/mL at stage 1′. Based on the available literature, we suggest that the potential effect of MLD on insulin levels may be due to the stimulation of the lymphatic system [32,59]. Recently, the presence of insulin-binding receptors in lymphoid endothelial cells (LECs) has been described [60]. In contrast, a 2018 study showed that LECs derived from human dermal lymphatic endothelial cells (HDLECs) exhibit significantly higher levels of insulin receptors (INSRs) compared to microvascular adipose endothelial cells [59].

According to the findings by Jaldin-Fincati et al., the presence of INSRs in lymphoid endothelial cells suggests that these cells may have important effects on the control of insulin levels in the interstitial space [59]. However, it is known that insulin level is closely associated with IR. Our team assessed the impact of MLD therapy on IR by referring to HOMA-IR. In patients of group I, we detected a statistically significant decrease in HOMA-IR, by 0.9, from 2.0 at stage 0′ to 1.1 at stage 1′. In patients of group II, we showed a statistically significant decrease in HOMA-IR by 0.7, from 2.6 at stage 0′ to 1.9 at stage 1′. In group III patients, we detected a decrease in HOMA-IR by 0.8, although not statistically significant, from 2.8 at stage 0′ to 2.0 at stage 1′. As in the case of the previously described parameters, in the control group, i.e., without MLD intervention, the HOMA-IR level did not change compared to the baseline status. It should be added that the HOMA-IR value in patients from groups I, II, and III at stage 0′ was above the reference value (>2.0). After 10 MLD sessions in groups I and II, the HOMA-IR value at stage 1′ decreased to the level of reference values. There is only one study in the research literature on the effect of manual therapy on the value of the HOMA-IR index in experimental animals [61]. The authors of this study evaluated the effect of abdominal massage on insulin levels and the HOMA-IR index in rats consuming a high-fat diet. The results showed a decrease in insulin levels and the HOMA-IR index in the group of rats consuming a high-fat diet that received abdominal massage, compared to the control group [61]. However, in recent years there have been more and more publications on the relationship between IR and the function of the lymphatic system [60,62]. Thus, Rehal et al. showed that weight gain by increasing the accumulation of reactive nitrogen species leads to dysfunction of the lymphatic system. In addition, they indicated that even short-term changes in lymphatic function can have a significant impact on carbohydrate metabolism, leading to IR [62]. Other authors, such as Freire de Oliveira et al., compared the effect of physical activity and MLD therapy on the function of the lymphatic system in postoperative patients [63]. In the group of active exercisers, an increase in the absorption of metabolites by the liver was shown, and patients in the MLD group had a significant increase in reflow through the skin [63]. The increase in backflow through the skin after the use of MLD observed in the work of Freire de Oliveira et al. [63] is one of the mechanisms that may explain the improvement in insulin sensitivity in our subjects. Finally, in our study, after MLD therapy, we observed a significant decrease in leptin concentration in group I patients, from 9.6 ng/mL at stage 0′ to 6.8 ng/mL, and in group II patients from 18.1 ng/mL at stage 0′ to 13 ng/mL at stage 1′. In group III patients, we detected a decrease in leptin concentration; however, it was not statistically significant—from 28.0 ng/mL at stage 0′ to 21.7 ng/mL at stage 1′. The positive effect of MLD on the concentration of leptin is related to the reports of Miller et al. [64]. Miller et al. attempted to identify the transport route of adipokines from adipose tissue to the bloodstream. Their findings showed that leptin (unlike adiponectin) is mainly transported via the lymphatic system [64]. We suggest that the improved function of the lymphatic system after MLD therapy may have an indirect effect on the increase in leptin transport. Interestingly enough, Sato et al. indicated leptin regulation as a viable strategy to improve lymphatic system function [28]. Sato et al. demonstrated that leptin levels regulate lymphatic vessel formation and cell proliferation in HDLECs [28]. In the available literature, no other studies have investigated the use of MLD on leptin levels in patients with abnormal body mass index. However, like in our study, other forms of non-pharmacological therapies, such as electroacupuncture [65], massage [66] and exercise [66,67], have been shown to reduce leptin levels in patients with abnormal body mass index. It should be added that in our group of patients from the control group (without MLD intervention), we observed an increase in leptin concentration by 1.3 ng/mL at stage 1′ compared to stage 0′. Based on our own research [14,32] and the available literature [68,69,70], we suggest that the use of MLD therapy may improve biochemical parameters, and MLD therapy may be related to the improvement of the lymphatic system’s function.

Our study provides additional evidence supporting the use of MLD as a preventive therapy, reducing the risk of obesity and IR. It seems that the inclusion of MLD as an additional method of supporting the treatment of people with abnormal body mass index, aimed at improving lymphatic function, is an innovative, non-pharmacological and effective solution. Finally, it should be highlighted that MLD is a non-invasive intervention, and so its use is safe. The search for interventions targeted at the lymphatic system is becoming an area of interest for more and more researchers and patients. In addition, our study provides new information regarding obesity and IR predictors, whose values could provide better reference ranges for use in preventive medicine. Several limitations of our study should be acknowledged. Firstly, due to the moderate size of our study and its non-randomized design, its findings should be verified in large, multicenter, randomized studies with long-term follow-up. Secondly, we investigated only selected biomarkers and metabolic parameters. Thirdly, the groups of our subjects were inhomogeneous in terms of gender. Finally, adequately powered randomized studies conducted in patients at risk are warranted to test whether MLD contributes to the prevention of obesity and insulin resistance.

## 4. Materials and Methods

### 4.1. Data Collection

Our study was conducted between January 2020 and September 2022. The subjects were recruited through the physiotherapy clinic and the Department and Clinic of Cardiology and Internal Medicine of the Institute of Maritime and Tropical Medicine in Gdynia, Poland, as well as via social media. One hundred patients participated in the study, 80 of whom were enrolled at the qualification stage, and 20 patients were excluded during the study. Ten patients were excluded due to the inability to participate in all stages for reasons related to infection, six for reasons related to the coronavirus disease 2019 (COVID-19) pandemic, and four for no reason provided. As a result, 60 patients completed all stages of the study. Based on medical records, history taking and biochemical parameters, we included patients who presented with carbohydrate-lipid metabolism disorders such as impaired glucose tolerance and hyperinsulinemia/insulin resistance. We excluded those with a history of diabetes mellitus, those taking medications that may modify glucose metabolism, those with uncontrolled hypertension, clinically significant arrhythmias, deep and superficial vein thrombosis, acute kidney injury, acute liver failure, malignancies and autoimmune and infectious diseases. MLD was considered to be contraindicated in those after surgery within the last six months, those with dermatitis or hematomas in the abdomen, groin and/or neck, and those who for any reason were unable to understand and/or follow instructions.

The patients were divided into four groups. Group I included 15 patients with a normal body mass index (BMI 22 ± 2 kg/m^2^, age 39 ± 13 years), group II included 15 overweight patients (BMI 28 ± 2 kg/m^2^, age 39 ± 11 years), and group III included 10 obese patients (BMI 34 ± 2 kg/m^2^, age 39 ± 11 years). Twenty subjects (subjects with a normal body mass index, overweight subjects, obese subjects; BMI 24 ± 4 kg/m^2^, age 39 ± 12 years) were included in group IV as controls.

Fourteen women and one man were included in group I, eleven women and four men were included in group II and nine women and one man were included in group III. The control group included twelve women and eight men.

All subjects received oral and written information about the study, and written informed consent forms were obtained prior to study inclusion.

The study was approved by the Bioethics Committee of the Medical University of Gdańsk (consent No. NKBBN/692/2019–2020; date of approval: 30 January 2020), and the study was conducted in accordance with the principles of the Declaration of Helsinki after the amendment in 2013.

### 4.2. Anthropometric and Blood Pressure Measurements

To determine BMI and waist–hip ratio (WHR) using the same scale, anthropometric measurements were performed in the patients of the study group and in the control group. To assess bioelectrical impedance, we used the Tanita SC-240 body composition analyzer (Tanita Corporation, Tokyo, Japan). During all measurements, the subject stood still, looking straight ahead, with feet together and arms at the sides. In addition, blood pressure was checked at each session with the physiotherapist.

The detailed procedures for anthropometric measurement, bioelectrical impedance assessment and blood pressure assessment have been described in detail in our previous publications [14,32].

### 4.3. Sample Collection and Laboratory Analyses

Following qualification, blood samples were collected to assess the concentration of FPG, 2h-PG, HbA1c, insulin, C-peptide, hsCRP, total cholesterol, high-density lipoprotein cholesterol (HDL-C), low-density lipoprotein cholesterol (LDL-C), triglycerides (TG), VEGF, interleukin-10 (IL-10), leptin and adiponectin. They were collected twice from each subject (at stage 0′ and stage 1′). Biochemical parameters were checked in patients from the study group at stage 0′ (before MLD therapy) and then at stage 1′ (one month after MLD therapy). The same tests in the case of those in the control group were carried out at stage 0′ and stage 1′, and the time frame between the collections at stage 0′ and 1′ was the same as in the study group.

**Figure 1:**
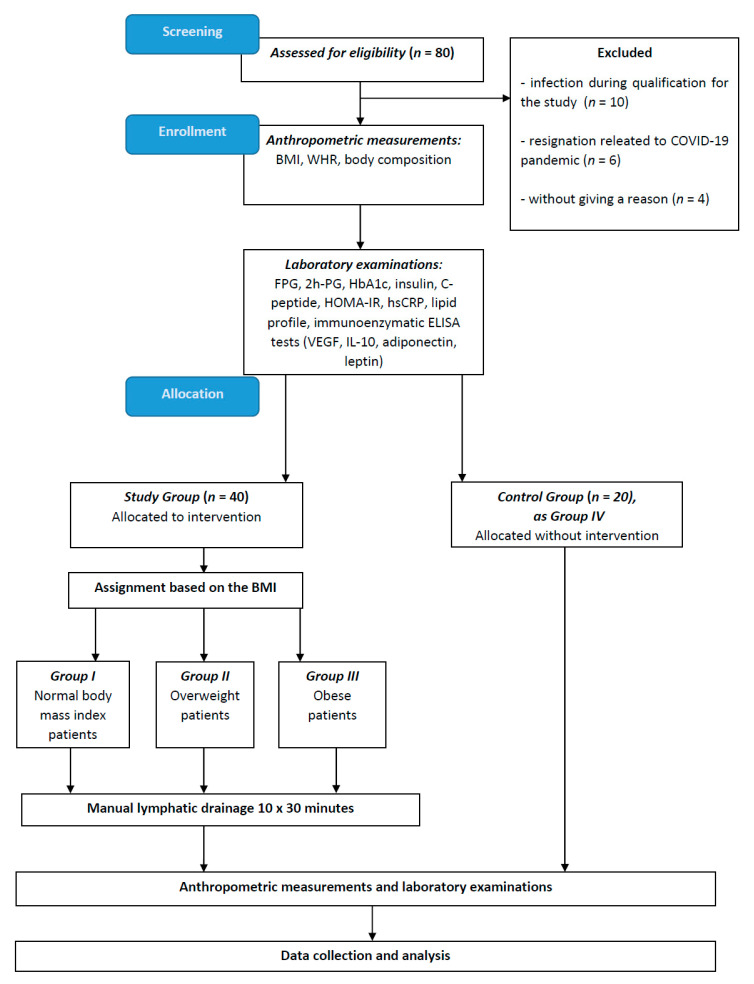
Study flow chart. Abbreviations: BMI, body mass index; coronavirus disease 2019, COVID-19; FPG, fasting plasma glucose; HbA1c, glycated hemoglobin; hsCRP, high-sensitivity C-reactive protein; HOMA-IR, homeostatic model assessment-insulin resistance; IL-10, interleukin-10; VEGF, vascular endothelial growth factor; WHR, waist–hip ratio; and 2h-PG, 2h-post-load glucose.

FPG and 2h-PG levels were determined by means of hexokinase and spectrophotometry (Cobas 8000 analyzer, Roche, Switzerland); HbA1c and hsCRP levels were determined using immunoturbidimetry (Cobas 8000 analyzer, Roche, Switzerland); and the level of C-peptide was determined by the ECLIA electrochemiluminescence method. In addition, total cholesterol, HDL-C, LDL-C and TG were determined with colorimetry (Cobas 8000, Roche, Switzerland) and insulin levels were determined using chemiluminescence (Cobas e601; Roche, Switzerland). Insulin resistance was assessed by calculating the homeostasis of the insulin resistance assessment model (HOMA-IR) [71] according to the following formula: HOMA-IR = (FPG × fasting serum insulin)/405, assuming <2 as normal in adults. The FPG and fasting serum insulin units for HOMA-IR calculation were mg/dL and µIU/mL, respectively. Blood samples taken to measure VEGF, IL-10 leptin and adiponectin levels were transported to the lab within 2 h; each sample was centrifuged at 2500 RPM for 15 min. The obtained blood serum was preserved in Eppendorf tubes (1.5 mL tubes, Eppendorf, Hamburg, Germany) and frozen at −80 °C until the day of testing. Adiponectin, leptin and VEGF, IL-10 levels were measured using ELISA enzyme immunoassay (R&D Systems, Minneapolis, MN, USA) according to the manufacturer’s protocol. The absorbance level was measured on an automatic plate reader (ChroMate 4300, Awareness Technology, Inc., Palm City, FL, USA). Reference curves were prepared according to the manufacturer’s recommendations. Detailed information about subject recruitment and the study methodology is presented in Figure 1.

### 4.4. Intervention MLD

Patients from the study group underwent MLD therapy. Each patient underwent 10 sessions of MLD therapy. They were held three times a week, for 30 min. A detailed description of the MLD that was used in our study patients was presented in previous manuscripts [14,32]. Briefly, the MLD we used can be demonstrated as follows: MLD according to Földi [72], which involved the abdominal cavity, groin area and the area of the venous angles. In the first stage, initial therapy was performed, including MLD in the suboccipital area, the area of the neck lymph nodes, and the supra- and subclavian fossae. In the next stage, MLD of the abdominal cavity was performed, including the area of the liver, stomach, large intestine of the ascending part, the transverse part and the descending part. In the last stage of MLD, deep abdominal and inguinal drainage was performed. During MLD therapy in the abdominal area, patients were asked to breathe deeply [72]. All patients of the study groups (group I, group II, group III) and the control group without MLD therapy (group IV) were advised to maintain their current lifestyle, and not to engage in any dietary programs or additional physical activities. MLD therapy was conducted by the same, experienced physiotherapist in all patients.

### 4.5. Statistical Analysis

The results were generated using OriginPro 2021 version 9.8 (OriginLab Corporation, Northampton, MA, USA). Normality was assessed using the Shapiro–Wilk test. Basic statistics, i.e., arithmetic mean and standard deviation (SD), were calculated for numerical values from a normally distributed population. Qualitative variables are presented in numerical and percentage form. To assess the strength and direction of the relationship between the two variables, a correlation analysis was performed with the calculation of the Pearson correlation coefficient. To determine the predictive value of individual clinical and biochemical parameters of obesity risk and IR, the Receiver Operating Curve (ROC) curves were used together with the Area Under Curve (AUC) analysis, as well as the determination of sensitivity and specificity, giving the cut-off value with the highest sensitivity and specificity. The ROC curve analysis calculations were performed at 0′ (before MLD therapy). The maximum area under the ROC curve (AUC_ROC_) (with values between 0 and 1) was a measure of the discriminatory power of the assay, and results are reported as means and 95% confidence interval (95% CI) [34,35]. One-way analysis of variance (ANOVA) was used to present changes in the biochemical parameters of patients in the study group and in the control group between stage 0 and 1. For each of the above-mentioned tests, a significance level of *p* ≤ 0.05 was assumed.

## 5. Conclusions

Our results indicate that MLD may have a positive effect on selected biochemical parameters, including insulin, 2h-PG, leptin and HOMA-IR, in normal weight and overweight patients. In addition, we identified cut-off values for leptin in the assessment of obesity and insulin in the assessment of insulin resistance in patients with abnormal body weight. Based on our findings, we hypothesize that MLD, when combined with caloric restriction and physical activity, may serve as an effective preventive intervention against the development of obesity and insulin resistance.

## Figures and Tables

**Table 1:** Clinical characteristics of patients with normal body mass index (group I), overweight patients (group II), obese patients (group III) and controls (group IV) without MLD therapy.

Parameter	All Patients with MLD Therapy (*n* = 60)	Group I Normal Body Mass Index Patients (*n* = 15)	Group II Overweight Patients (*n* = 15)	Group III Obese Patients (*n* = 10)	Group IV as Control/ without MLD Therapy (*n* = 20)	*p*	
Age [years]	39 ± 11	39 ± 13	39 ± 11	39 ± 11	39 ± 12	I vs. II	ns
						I vs. III	ns
						I vs. IV	ns
						II vs. III	ns
						II vs. IV	ns
						III vs. IV	ns
SBP [mmHg]	130 ± 8	121 ± 2	126 ± 3	140 ± 4	130 ± 5	I vs. II	ns
						I vs. III	0.000000007 *
						I vs. IV	ns
						II vs. III	0.0006 *
						II vs. IV	ns
						III vs. IV	0.00000008 *
DBP [mmHg]	82 ± 5	74 ± 6	81 ± 5	87 ± 7	84 ± 5	I vs. II	ns
						I vs. III	ns
						I vs. IV	ns
						II vs. III	ns
						II vs. IV	ns
						III vs. IV	ns
BMI [kg/m^2^]	27 ± 5	22 ± 2	28 ± 2	34 ± 2	24 ± 4	I vs. II	0.018 *
						I vs. III	0.015 *
						I vs. IV	ns
						II vs. III	0.0001 *
						II vs. IV	0.002 *
						III vs. IV	0.000000005 *
WHR	0.9 ± 0.2	0.8 ± 0.06	1 ± 0.04	1.2 ± 0.08	0.8 ± 0.09	I vs. II	0.00001 *
						I vs. III	0.0000003 *
						I vs. IV	ns
						II vs. III	0.0000002 *
						II vs. IV	0.00009 *
						III vs. IV	0.00000009 *
VAT [LVL]	6 ± 4	4 ± 2	7 ± 3	9 ± 2	5 ± 3	I vs. II	0.002 *
						I vs. III	0.005 *
						I vs. IV	ns
						II vs. III	0.008
						II vs. IV	ns
						III vs. IV	0.0003 *

Data are presented as mean values and their standard deviations; * *p*-value—significant difference (*p* < 0.05). Abbreviations: BMI, body mass index; DBP, diastolic blood pressure; SBP, systolic blood pressure; VAT, visceral adipose tissue; WHR, waist–hip ratio; I vs. II (group I vs. group II); I vs. III (group I vs. group III); I vs. IV (group I vs. group IV); II vs. III (group II vs. group III), II vs. IV (group II vs. group IV); and III vs. IV (group III vs. group IV).

**Table 2:** Relationship between biochemical parameters and BMI, WHR and VAT of subjects from the study group and control subjects.

Parameter	BMI [kg/m^2^] (*n* = 60)	WHR (*n* = 60)	VAT [LVL] (*n* = 60)
FPG [mg/dL]	*r* = 0.222	*r* = 0.284	*r* = 0.493
	*p* = 0.167	*p* = 0.756	*p* = 0.001 *
2h-PG [mg/dL]	*r* = 0.321	*r* = 0.303	*r* = 0.434
	*p* = 0.043 *	*p* = 0.056	*p* = 0.005 *
HbA1c [%]	*r* = 0.379	*r* = 0.384	*r* = 0.444
	*p* = 0.142	*p* = 0.141	*p* = 0.004 *
Insulin [µIU/mL]	*r* = 0.465	*r* = 0.433	*r* = 0.396
	*p* = 0.002 *	*p* = 0.005 *	*p* = 0.005 *
C-peptide [ng/mL]	*r* = 0.471	*r* = 0.447	*r* = 0.322
	*p* = 0.002 *	*p* = 0.003 *	*p* = 0.042 *
HOMA-IR	*r* = 0.433	*r* = 0.462	*r* = 0.410
	*p* = 0.005 *	*p* = 0.002 *	*p* = 0.008 *
hsCRP [mg/L]	*r* = 0.234	*r* = 0.779	*r* = 0.242
	*p* = 0.145	*p* = 0.004 *	*p* = 0.132
Total cholesterol [mg/dL]	*r* = 0.999	*r* = 0.909	*r* = 0.757
	*p* = 0.001 *	*p* = 0.018 *	*p* = 0.050 *
HDL-C [mg/dL]	*r* = −0.325	*r* = −0.211	*r* = −0.490
	*p* = 0.040*	*p* = 0.190	*p* = 0.001 *
LDL-C [mg/dL]	*r* = 0.990	*r* = 0.709	*r* = 0.757
	*p* = 0.001 *	*p* = 0.018 *	*p* = 0.017 *
TG [mg/dL]	*r* = 0.291	*r* = 0.316	*r* = 0.500
	*p* = 0.067	*p* = 0.046 *	*p* = 0.001 *
VEGF [pg/mL]	*r* = 0.998	*r* = 0.716	*r* = 0.868
	*p* = 0.0002 *	*p* = 0.059 *	*p* = 0.027 *
IL-10 [pg/mL]	*r* = 0.628	*r* = 0.438	*r* = 0.696
	*p* = 0.078	*p* = 0.125	*p* = 0.063
Adiponectin [μg/mL]	*r* = 0.683	*r* = 0.531	*r* = 0.810
	*p* = 0.066	*p* = 0.101	*p* = 0.039 *
Leptin [ng/mL]	*r* = 0.718	*r* = 0.733	*r* = 0.581
	*p* = 0.0000001 *	*p* = 0.00000007 *	*p* = 0.00005 *

Data are presented as the Pearson correlation coefficient; * *p*-value—significant difference (*p* < 0.05). Abbreviations: BMI, body mass index; FPG, fasting plasma glucose; HbA1c, glycated hemoglobin; HDL-C, high density lipoprotein cholesterol; HOMA-IR, Homeostatic Model Assessment–Insulin Resistance; hsCRP, high-sensitivity C-reactive protein; IL-10, interleukin-10; LDL-C, low-density lipoprotein cholesterol; TG, triglycerides; VAT, visceral adipose tissue; VEGF, vascular endothelial growth factor; WHR, waist–hip ratio; and 2h-PG, 2h-post-load glucose.

**Table 3:** AUCROC analysis of the biochemical parameters studied in patients from the study and control groups for obesity development.

Variable	AUC	95% CI for AUC	Specificity	Sensitivity	Optimal Cut-Off	*p*-Value
FPG	48.65%	32.86–64.66%	43.43%	51.60%	98.5 [mg/dL]	0.08
2h-PG	65.2%	47.21–83.18%	29%	58%	106.5 [mg/dL]	0.111
HbA1c	42.74%	26.92–58.56%	53.64%	53.40%	5.2 [%]	0.369
Insulin	81.51%	69.23–93.79%	32.45%	62.13%	9.5 [µIU/mL]	0.00009 *
C-peptide	80.68%	68.73–92.62%	42.39%	64.53%	2.3 [ng/mL]	0.0001 *
HOMA-IR	79.97%	67.16–92.78%	33.16%	69.53%	1.8	0.0002 *
Total cholesterol	66.30%	49.61–82.99%	42.98%	55.47%	195 [mg/dL]	0.043 *
HDL-C	37.42%	21.86–52.97%	57.57%	41.09%	59.5 [mg/dL]	0.119
LDL-C	43.58%	28.59–58.57%	64.5%	48.83%	68 [mg/dL]	0.426
TG	64.44%	49.49–79.38%	39.11%	57.7%	96.5 [mg/dL]	0.073
hsCRP	68.22%	53.62–82.33%	16.28%	38.09%	3.4 [mg/L]	0.024 *
VEGF	44.92%	28.75–61.10%	51.88%	48.47%	215.3 [pg/mL]	0.530
IL-10	70.8%	53.75–87.84%	30.4%	41.33%	0.35 [pg/mL]	0.029 *
Adiponectin	41.46%	25.65–57.27%	53.53%	47.36%	3.5 [μg/mL]	0.290
Leptin	82.79%	71.51–94.08%	41.92%	27.22%	17.7 [ng/mL]	0.00004 *

* *p*-value—significant difference (*p* < 0.05). Abbreviations: AUC, area under the curve; CI, confidence interval; FPG, fasting plasma glucose; HbA1c, glycated hemoglobin; HDL-C, high-density lipoprotein cholesterol; HOMA-IR, Homeostatic Model Assessment–Insulin Resistance; hsCRP, high-sensitivity C-reactive protein; IL-10, interleukin-10; LDL-C, low-density lipoprotein cholesterol; TG, triglycerides; VEGF, vascular endothelial growth factor; and 2h-PG, 2h-post-load glucose.

**Table 4:** AUCROC analysis of the studied biochemical parameters in patients from the study and control group for obesity development.

Variable	AUC	95% CI for AUC	Specificity	Sensitivity	Optimal Cut-Off	*p*-Value
FPG	59.78%	43.01–76.56%	45.67%	53.53%	89.5 [mg/dL]	0.232
2h-PG	70.93%	53.92–87.94%	41.41%	60.78%	93 [mg/dL]	0.028 *
HbA1c	59.98%	43.46–76.50%	51.25%	58.77%	5.2 [%]	0.223
Insulin	93.05%	85.15–100%	36.9%	78%	6.9 [µIU/mL]	0.053 *
C-peptide	89.35%	78.93–99.77%	40.79%	77.56%	1.8 [ng/mL]	0.000001 *
Total cholesterol	77.31%	63.82–90.80%	42.48%	67.75%	198 [mg/dL]	0.0008 *
HDL-C	41.53%	25.67–57.39%	50.84%	42.15%	59.5 [mg/dL]	0.301
LDL-C	41.40%	25.23–57.57%	52.18%	43.51%	72 [mg/dL]	0.294
TG	69.31%	53.67–84.94%	43.98%	62.89%	95 [mg/dL]	0.018 *
hsCRP	68.71%	54.47–54.47%	20.34%	39.39%	1.9 [mg/L]	0.022 *
VEGF	49.53%	32.66–66.41%	49.60%	49.07%	208.1 [pg/mL]	0.954
IL-10	47.60%	28.98–66.21%	35.6%	32.66%	0.85 [pg/mL]	0.801
Adiponectin	42.72%	24.93–60.51%	52.21%	44.99%	3.6 [μg/mL]	0.374
Leptin	79.76%	66.30–93.21%	39.77%	69.44%	17.6 [ng/mL]	0.0002 *

* *p*-value—significant difference (*p* < 0.05). Abbreviations: AUC, area under the curve; CI, confidence interval; FPG, fasting plasma glucose; HbA1c, glycated hemoglobin; HDL-C, high-density lipoprotein cholesterol; hsCRP, high-sensitivity C-reactive protein; IL-10, interleukin-10; LDL-C, low-density lipoprotein cholesterol; TG, triglycerides; VEGF, vascular endothelial growth factor; and 2h-PG, 2h-post-load glucose.

**Table 5:** Concentrations of investigated biochemical parameters at stage 0′ and 1′ in the group of patients with a normal body mass index (group I), overweight patients (group II), obese patients (group III) and people in the control group (without MLD therapy).

Parameter		Group I (*n* = 15)	Group I *p* 0′–1′	Group II (*n* = 15)	Group II *p* 0′–1′	Group III (*n* = 10)	Group III *p* 0′–1′	Group IV (*n* = 20)	Group IV *p* 0′–1′
FPG [mg/dL]	0′	85 ± 8	0.814	97 ± 5	0.254	95 ± 22	0.671	90 ± 7	0.899
	1′	86 ± 10		92 ± 5		91 ± 15		92 ± 7	
2h-PG [mg/dL]	0′	79 ± 15	0.736	119 ± 7	0.049 *	103 ± 31	0.998	100 ± 3	0.726
	1′	77 ± 15		100 ± 5		103 ± 28		102 ± 3	
HbA1c [%]	0′	5.2 ± 0.5	0.751	5.2 ± 0.3	0.999	5.3 ± 0.6	0.665	5.2 ± 0.5	0.986
	1′	5.2 ± 0.5		5.2 ± 0.3		5.3 ± 0.5		5.2 ± 0.5	
Insulin [µIU/mL]	0′	8 ± 2	0.039 *	8 ± 4	0.899	9 ± 4	0.670	6 ± 4	0.968
	1′	5 ± 2		9 ± 4		10 ± 4		6 ± 3	
C-peptide [ng/mL]	0′	1.9 ± 1.8	0.240	2 ± 0.8	0.878	2.2 ± 0.8	0.813	1.6 ± 0.5	0.865
	1′	1.3 ± 0.3		2 ± 0.7		2.1 ± 0.8		1.7 ± 0.5	
hsCRP [mg/L]	0′	1 ± 0.1	0.998	4 ± 6.6	0.275	2 ± 2.4	0.899	2 ± 1.4	0.897
	1′	1 ± 0.2		2 ± 1.5		3 ± 3.8		2 ± 1.3	
Total cholesterol [mg/dL]	0′	193 ± 34	0.881	208 ± 47	0.914	204 ± 7	0.661	178 ± 35	0.888
	1′	194 ± 31		206 ± 47		208 ± 5		175 ± 36	
HDL-C [mg/dL]	0′	66 ± 10	0.712	58 ± 16	0.929	57 ± 15	0.942	62 ± 15	0.713
	1′	64 ± 9		58 ± 17		57 ± 15		59 ± 15	
LDL-C [mg/dL]	0′	111 ± 28	0.674	120 ± 41	0.961	124 ± 20	0.912	98 ± 29	0.869
	1′	116 ± 29		120 ± 41		130 ± 25		96 ± 32	
TG [mg/dL]	0′	78 ± 28	0.642	158 ± 32	0.901	114 ± 71	0.840	89 ± 46	0.856
	1′	73 ± 24		153 ± 30		108 ± 46		93 ± 40	
HOMA-IR	0′	2 ± 2	0.003 *	2.6 ± 0.9	0.049 *	2.8 ± 1.5	0.169	1.4 ± 0.8	0.960
	1′	1.1 ± 0.4		1.9 ± 0.7		2 ± 1		1.4 ± 0.6	
VEGF [pg/mL]	0′	252 ± 241	0.687	217 ± 137	0.598	230 ± 169	0.688	277 ± 181	0.803
	1′	218 ± 215		191 ± 125		255 ± 105		290 ± 155	
IL-10 [pg/mL]	0′	0.5 ± 0.5	0.464	0.3 ± 0.2	0.807	0.7 ± 0.9	0.252	0.6 ± 0.3	0.822
	1′	0.3 ± 0.3		0.3 ± 0.1		0.3 ± 0.01		0.6 ± 0.3	
Adiponectin [μg/mL]	0′	3.3 ± 1.0	0.883	2.9 ± 1.1	0.495	3.6 ± 2.5	0.975	3.6 ± 2.5	0.975
	1′	3.3 ± 1.2		2.7 ± 1.1		3.6 ± 2.9		3.6 ± 2.9	
Leptin [ng/mL]	0′	9.6 ± 1.9	0.041 *	18.1 ± 2.1	0.033 *	28.0 ± 12.1	0.206	20.0 ± 10.5	0.823
	1′	6.8 ± 1.4		13.0 ± 2.5		21.7 ± 9.2		21.3 ± 12.8	

Data are presented as mean values and their standard deviations; * *p*-value—significant difference (*p* < 0.05). Abbreviations: FPG, fasting plasma glucose; HbA1c, glycated hemoglobin; HDL-C, high-density lipoprotein cholesterol; HOMA-IR, Homeostatic Model Assessment–Insulin Resistance; hsCRP, high-sensitivity C-reactive protein; IL-10, interleukin-10; LDL-C, low-density lipoprotein cholesterol; TG, triglycerides; VEGF, vascular endothelial growth factor; 2h-PG, 2h-post-load glucose; 0′, before therapy; and 1′, one month after MLD therapy.

## Data Availability

The data presented in this study are available on request from the corresponding author: klaudia.antoniak@gumed.edu.pl.

## Abbreviations

BMI	body mass index
COVID-19	coronavirus disease 2019
eNOS	endothelial nitric oxide synthase
FFA	free fatty acids
FPG	fasting plasma glucose
GLUT4	glucose transporter type 4
HbA1c	glycated hemoglobin
HDL-C	high-density lipoprotein cholesterol
HOMA-IR	Homeostatic Model Assessment-Insulin Resistance
HDLECs	human dermal lymphatic endothelial cells
hsCRP	high-sensitivity C-reactive protein
IDF	International Diabetes Federation
IL-10	interleukin-10
INSRs	insulin receptors
IR	insulin resistance
LDL-C	low-density lipoprotein cholesterol
LECs	lymphoid endothelial cells
MLD	manual lymphatic drainage
TG	triglycerides
T2DM	type 2 diabetes mellitus
VAT	visceral adipose tissue
VEGF	vascular endothelial growth factor
WAT	white adipose tissue
WHR	waist–hip ratio
2h-PG	2h-post-load glucose
